# Anoikis resistance––protagonists of breast cancer cells survive and metastasize after ECM detachment

**DOI:** 10.1186/s12964-023-01183-4

**Published:** 2023-08-03

**Authors:** Yalan Dai, Xinyi Zhang, Yingjun Ou, Linglin Zou, Duoli Zhang, Qingfan Yang, Yi Qin, Xiuju Du, Wei Li, Zhanpeng Yuan, Zhangang Xiao, Qinglian Wen

**Affiliations:** 1 Department of Oncology, The Affiliated Hospital of Southwest Medical University, Luzhou, China; 2 Department of Oncology, Garze Tibetan Autonomous Prefecture People’s Hospital, Kangding, China; 3 School of Biomedical Sciences, The Chinese University of Hong Kong, Shenzhen, China; 4 Clinical Medicine School, Southwest Medicial Univercity, Luzhou, China; 5 Orthopaedics, Garze Tibetan Autonomous Prefecture People’s Hospital, Kangding, China; 6 Laboratory of Molecular Pharmacology, Department of Pharmacology, School of Pharmacy, Southwest Medical University, Luzhou, China; 7 Southwest Medical University, Luzhou, China

**Keywords:** Anoikis, Anoikis resistance, Breast cancer, ECM detachment

## Abstract

Breast cancer exhibits the highest global incidence among all tumor types. Regardless of the type of breast cancer, metastasis is a crucial cause of poor prognosis. Anoikis, a form of apoptosis initiated by cell detachment from the native environment, is an outside-in process commencing with the disruption of cytosolic connectors such as integrin-ECM and cadherin-cell. This disruption subsequently leads to intracellular cytoskeletal and signaling pathway alterations, ultimately activating caspases and initiating programmed cell death. Development of an anoikis-resistant phenotype is a critical initial step in tumor metastasis. Breast cancer employs a series of stromal alterations to suppress anoikis in cancer cells. Comprehensive investigation of anoikis resistance mechanisms can inform strategies for preventing and regressing metastatic breast cancer. The present review first outlines the physiological mechanisms of anoikis, elucidating the alterations in signaling pathways, cytoskeleton, and protein targets that transpire from the outside in upon adhesion loss in normal breast cells. The specific anoikis resistance mechanisms induced by pathological changes in various spatial structures during breast cancer development are also discussed. Additionally, the genetic loci of targets altered in the development of anoikis resistance in breast cancer, are summarized. Finally, the micro-RNAs and targeted drugs reported in the literature concerning anoikis are compiled, with keratocin being the most functionally comprehensive.

Video Abstract

**Supplementary Information:**

The online version contains supplementary material available at 10.1186/s12964-023-01183-4.

## Introduction

The World Health Organization's International Agency for Research on Cancer (IARC) posted the latest data on global cancer burden in 2020. Revealing a rapid increase in breast cancer (BC) cases to 2.26 million. This accounted for approximately 11.7% of all new cancer patients, establishing BC as the most prevalent cancer worldwide [1]. BC exhibits significant heterogeneity, with some cases presenting slow growth and favorable prognosis, while others are highly aggressive and rapidly metastasize to other organs [2].

Molecular subtypes of BC include Luminal A, Luminal B, human epidermal growth factor receptor2 (HER2) overexpression, normal breast-like, and Basal-like or Triple Negative cancer (TNBC) [3]. This molecular sub-classification could improve BC treatment techniques to a large extent, including guiding the delivery of targeted therapies such as hormone therapy (e.g., Toremifene) and HER2-targeted therapy (e.g., Pertuzumab) [4]. Intrinsic subtypes of BC have been used in research settings for more than two decades [5], with ongoing efforts to refine differentiation among BC subtypes [6]. Evidence suggests that BC cells may interconvert between different disease subtypes, indicating the potential coexistence of multiple BC subtypes within a single tumor [7]. Regardless of the subtypes, metastasis significantly reduces patients’ survival rate [8]. A statistical analysis of all BC patients diagnosed in the US between 2009 and 2015 showed 5-year survival rates of 98% for stage I, 92% for stage II, 75% for stage III and 27% for stage IV [9].

**Fig. 1:**
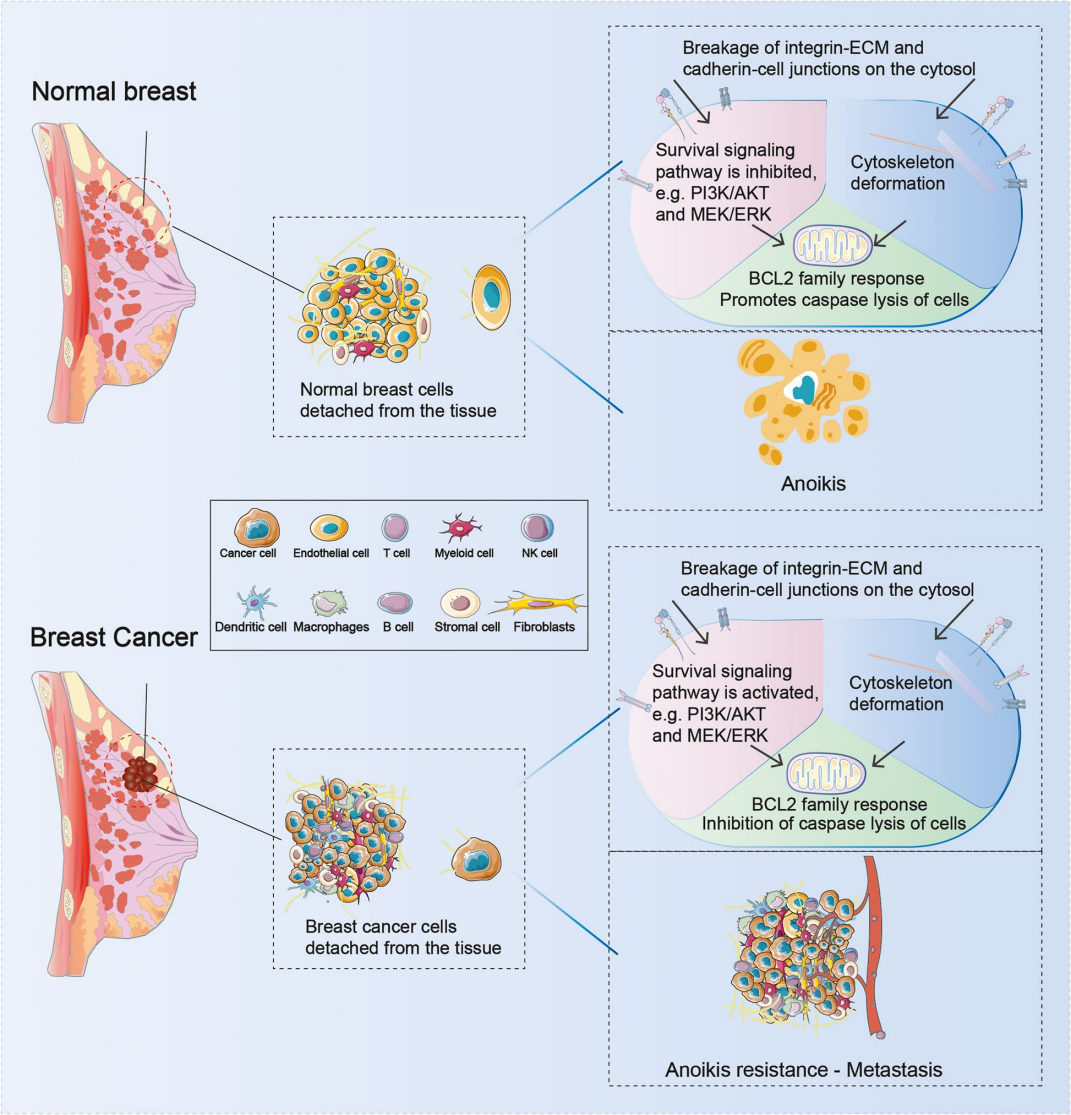
When normal breast cells are detached from their natural environment, this triggers changes in the corresponding structures in the cell membrane, which, by inhibiting intracellular pro-survival signalling pathways and triggering changes in the cytoskeleton, eventually causes the cells to undergo physiological anoikis. When breast cancer cells are detached from tumour tissue, the cells are altered from receptors on the cell membrane, resulting in significant activation of the pro-survival pathway and changes to the cytoskeleton, allowing the tumour cells to develop anoikis resistance after detachment from their native environment, thus allowing them to metastasise and continue to grow elsewhere

Metastasis initiation occurs when local tumor cells detach from the extracellular matrix(ECM) at the site of origin [10]. Loss of contact with the ECM or other cells induces anoikis, a specific form of programmed cell death and a subtype of apoptosis [11]. Anoikis is a pivotal mechanism to inhibit cell colonization and growth in the new stromal environment [12]. Tumor cells develop a survival phenotype that allows them to bypass anoikis upon ECM detachment, migrate to other organs, and repopulate to form metastatic tumors (Fig. 1) [13]. Anoikis resistance is a prerequisite and a significant indicator of tumor cell metastatic potential. Preserving anoikis functional integrity is an essential means of preventing metastasis, necessitating a deeper understanding of tumor cell resistance mechanisms to anoikis [14].

Studying anoikis resistance mechanisms in BC is important because mammary gland epithelial cells adhere to laminin-rich basement membranes via integrins, rather than interacting with collagen I(COLI) [15]. Catheter space filling is a feature of many early BC lesions [16], and anoikis resistance facilitates vitro 3D ductal filling in breast follicular epithelial structures [17], which is a critical process in BC distant metastasis.

Overall, anoikis is a specific form of programmed cell death induced by the loss of cell contact with the extracellular matrix and other cells, and a subtype of apoptosis that still induces cell death via the traditional apoptotic pathway. This review examines the activation of anoikis by adhesion-initiating signals in both normal and metastatic tumor cells, which trigger a series of changes in intracellular pathways, proteins, cytoskeleton, and genetic material. A comprehensive understanding of anoikis resistance mechanisms in BC cells may provide potential opportunities for the prevention and treatment of metastatic cancer.

## Main text

### Physiological anoikis (Fig. 2)

**Fig. 2:**
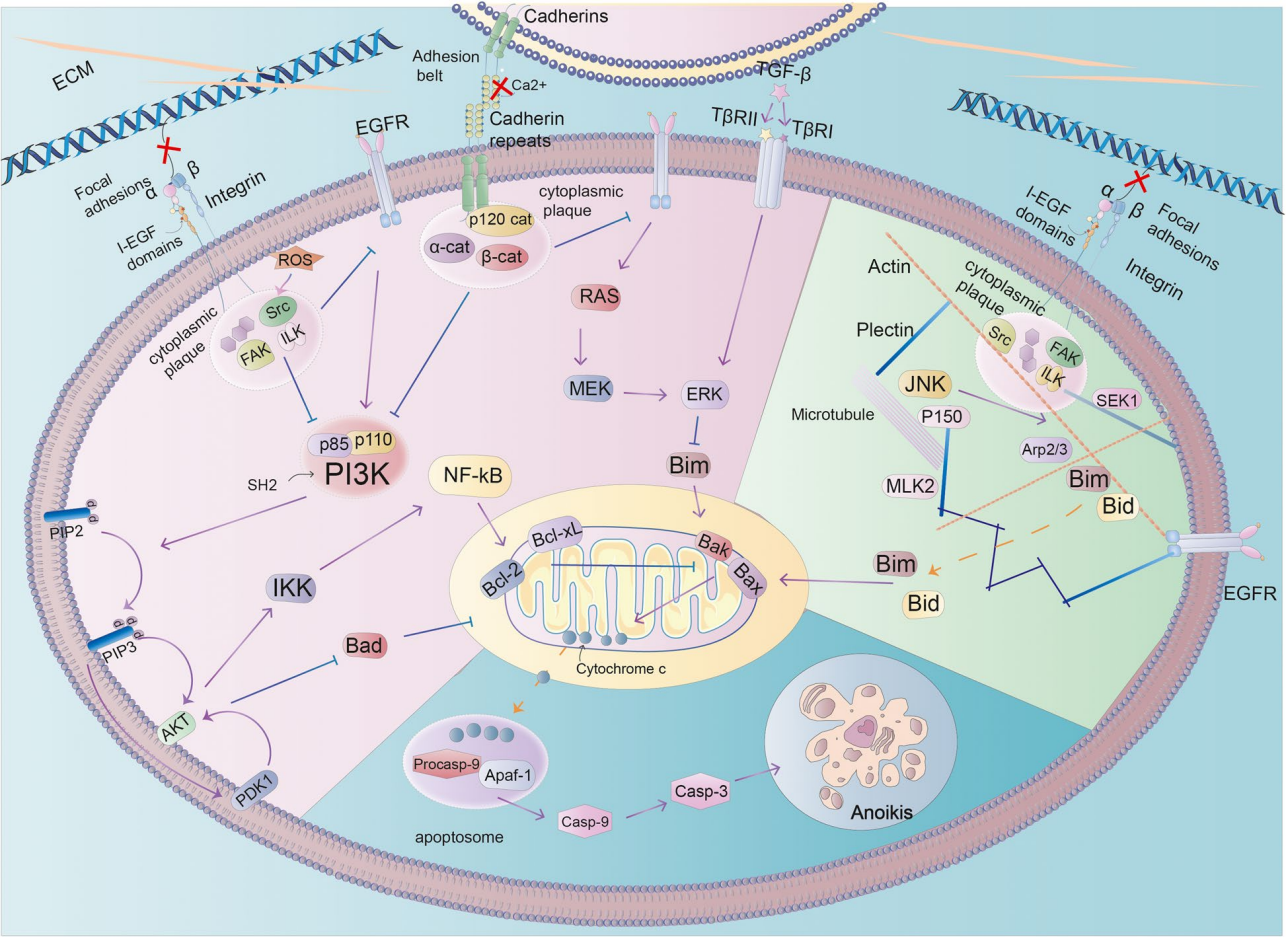
Detachment of normal breast cells from their native environment triggers activation of adhesion mediators on the cell membrane, inhibition of survival pathways (e.g., PI3K-AKT, MEK-ERK), and simultaneous alteration of the cytoskeletal structure, triggering a BCL2 family protein response, activation of the mitochondrial pathway of apoptosis, and ultimately the release of caspases, triggering anpikis

#### ECM-The cradle of cell growth

ECM provides adhesion support for cells and regulates cellular physiological behavior through signaling [18]. ECM detachment causes a range of metabolic changes, including glucose uptake defective, the pentose phosphate pathway(PPP) flux decreases, cellular ATP levels lower, and reactive oxygen species (ROS) increases [19]. Proteolytic cleavage, integral proteins, or changes in microenvironment can also activate potential TGF-β [20], activating classical Smad signaling [21], and non-Smad signaling pathways including MAP/Erk, TβRI induced Shc phosphorylation, as well as Ras, Rho, Rac, and CDC42 small GTases, further promote anoikis development [22].

### Cell membrane alterations

**Fig. 3:**
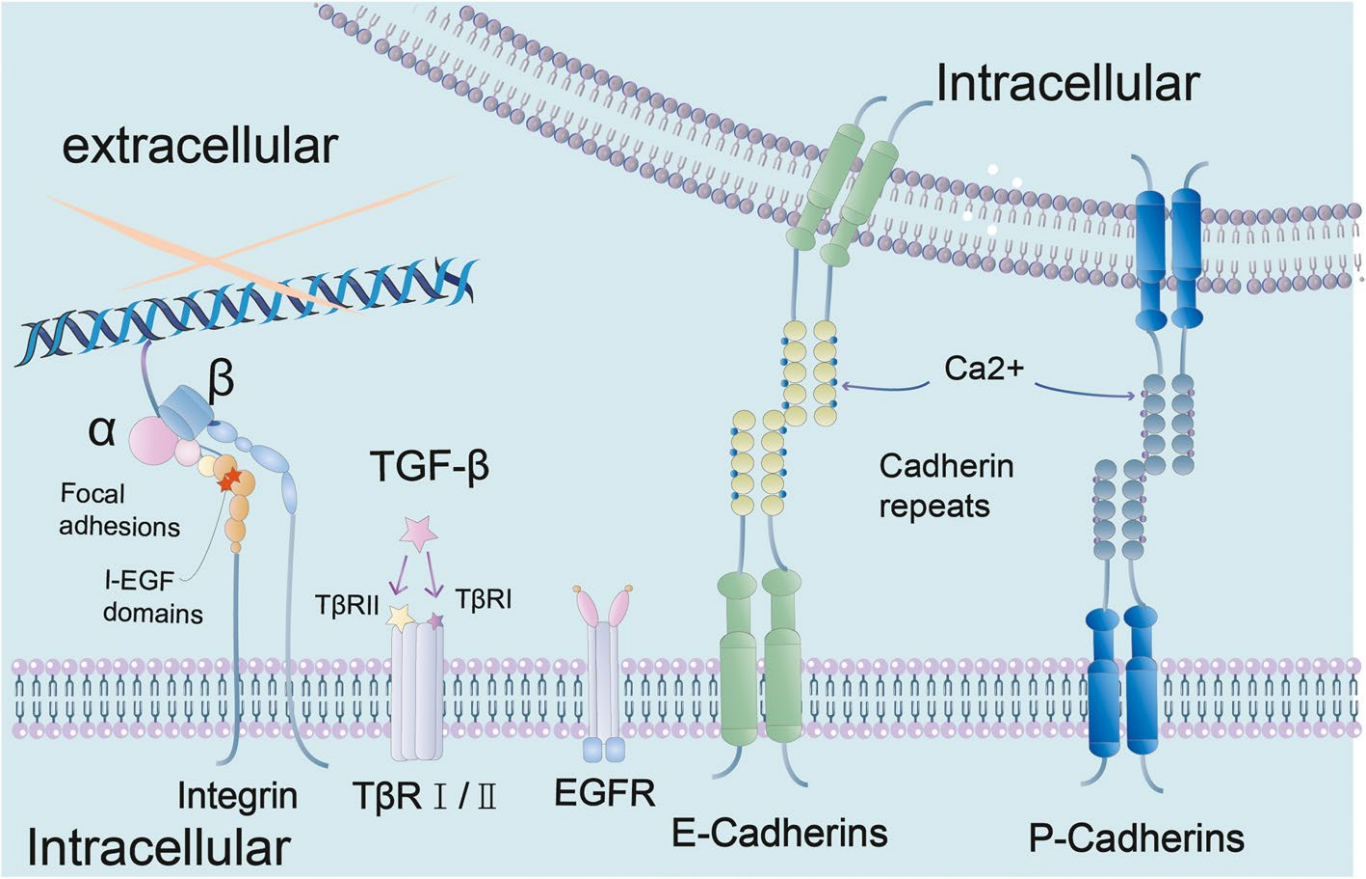
CAMs associated with anoikis are described here, where integrins mediate cell-ECM adhesion, E-cadherin and P-cadherin mediate cell–cell adhesion, and some cell membrane receptors such as TβR I/II and EGFR also affect anoikis when cells lose their adhesion

Cells express various cell adhesion molecules (CAM) that mediate cell–cell or cell-ECM contacts (Fig. 3) [23]. The cell-ECM linkage is a focal adhesion, which relies on integral protein-actin interactions [24]. The adhesion belt is responsible for cell–cell contacts, with the main proteins involved in adhesion band junctions being cadherin and actin [25]. These CAMs are typically transmembrane proteins consisting of three structural domains: an extracellular structural domain responsible for ligand binding, a transmembrane structural domain, and a cytoplasmic tail attached to the actin cytoskeleton by a protein complex such as an enzyme or kinase [26]. Many transmembrane proteins, such as growth factor receptor (GFR), are also present in the cell membrane and are subject to changes in cell-ECM or cell–cell contact status [27].

### Integrin-messengers of cell and ECM

Integrins are transmembrane αβ heterodimers [28]. These cell surface glycoproteins mediate cell-ECM interactions and dynamic adhesion [29]. The α and β subunits of integrins typically include a large extracellular structural domain, a transmembrane helix, and a short cytoplasmic tail [30].

The extracellular structural domain of integrins binds to specific ECM proteins, such as collagen, vitronectin, fibronectin, and other proteins [29]. Disassociation of cells from ECM can lead to the activation of integrins, resulting in a shift of integrin legs from an inactive leaning-together state to an active elongated detached state [31]. Long-range conformational changes in extracellular structural domains cause intracellular protein reactions that trigger the reorganization of ligand binding sites [32]. Through the recruitment and aggregation of integrins, intracellular proteins bind directly or indirectly to integrin tails, forming a specific structure of focal adhesion [33]. Integrin tails do not have intrinsic enzymatic activity. However, adherent spots contain many protein kinases and scaffolding proteins, and some adherent spot proteins (e.g., talin) can bind actin and thus have specific effects [34].

The intracellular signaling pathway triggered by integral proteins has two main functions: to organize the actin cytoskeleton and to regulate cellular behavior [35]. Integrins regulate the cytoskeleton by directly binding actin proteins, including synuclein, nuclein and filament proteins [36]. Integrins activate their regulated signaling pathways by phosphorylating integrin-related kinase (ILK), proto-oncogene tyrosine protein kinase (Src) and focal adhesion kinase (FAK), thereby regulating cellular behavior [37].

### Cadherin-messengers of cells and cells

Cadherins are responsible for cell–cell adhesion and include type I, II, and III/atypical cadherins, all of which are expressed in the mammary gland [38]. The most important E-cadherin is a type I cadherin, a membrane glycoprotein located at the cell adhesion junctions, which anchors epithelial cells to each other and is essential for the adhesion of adjacent epithelial cells [39]. In normal breasts, monolayer epithelial cap cells of terminal buds also express P-cadherin, which is important to the branching process of breast ducts [40]. At the adhesion junctions, the cadherin cytoplasmic tail provides binding sites for p120-, α-, γ-, and β-catenin, facilitating connections between the signaling pathways and actin cytoskeleton [41]. Anoikis is induced by cadherin when cell–cell adhesion is broken.

### Epidermal Growth Factor Receptor (EGFR)

In human mammary epithelial cells, ECM exposure is the main factor in EGFR expression and downstream signaling activation [42]. Activated EGFRs implicate downstream molecules in the response, such as Janus-activated kinase (JAK), Ras, phospholipase Cγ (PLCγ) and phosphatidylinositol 3-kinase (PI3K) [43]. EGFR and integrins are functionally coupled [44]. Loss of integrin-ECM adhesion leads to downregulation of EGFR expression and inhibition of downstream EGFR molecules, [42], which synergically induced anoikis.

### Cytoplasm

ECM detachment in mammary epithelial cells promotes changes in CAM and protein receptors on the cell membrane, resulting in reduced intracellular EGFR, PI3K/Akt and Mek/Erk signals, which are transmitted to the mitochondria and affect cell survival [45]. The mitochondria play a critical role in anoikis [46]. The mitochondrial intermembrane space (IMS) contains many key pro-apoptotic factors, such as cytochrome c. When incoming survival signals to the mitochondria are reduced, pro-apoptotic factors are released into the cytoplasm, thereby triggering anoikis [47].

### Cytoplasmic plaque

The cytoplasmic region contains several cytoplasmic patches, which are multimolecular protein complexes. Cytoplasmic plaques are involved in building membrane protein scaffolds and cytoskeletons, regulating polarity, and transmitting signals [48]. Cytoplasmic plaque components of the cell-ECM and cell–cell are each distinct.

### Focal adhesion-cytoplasmic plaque

Focal adhesion is the cytoplasmic part of integrins and the site of proteoglycan-mediated adhesion to the actin cytoskeleton [34]. It contains various kinases, such as focal adhesion kinase(FAK) and Src, and serine/threonine kinase ILK [49]. FAK is a multifunctional protein that integrates signals sensed by integrin or growth factor receptors, which are then transduced into the cel [50]. Src interacts with FAK and facilitates FAK phosphorylation and activation [51]. ILK connects integrins to the actin cytoskeleton by interacting with pilings and parvins [52]. It also binds to phosphatidylinositol 3,4,5-triphosphate(PIP3) and affects the downstream signaling pathway [53].

### Adhesion belt-cytoplasmic plaque

The intracellular structural domain of cadherin is linked to actin fibers through protein-mediated connection in the cytoplasmic plaque, which contains β-catenin, α-catenin, p120-catenin, etc. [54]. P120-catenin is a substrate for Src and several receptor tyrosine kinases, and interacts with the proximal membrane domain of cadherin to direct cadherin aggregation and cell motility [55]. β-catenin binds to α-catenin, which connects cadherin to actin cytoskeleton [56]. The cadherin protein linker complex enhances adhesion by linking α-catenin to actin cytoskeleton. α-catenin in its monomeric form binds to calmodulin-linkerin complex via β-catenin, while the homodimeric form of α-catenin binds to F-actin [57]. Cadherin regulates the expression of GFR, PI3K and ERBB4 through catenins [58].

### Signaling pathway

#### PI3K-AKT signaling pathway

FAK, cadherin and EGFR are all upstream activation conditions of PI3K [59]. PI3K phosphorylates the plasma membrane lipid substrate PIP2, generating PIP3 [60]. PIP3 then recruits AKT and 3-phosphatidylinositol-dependent protein kinase 1 (PDK1) to the plasma membrane via the PH structural domain, phosphorylating AKT and PDK1 at Ser and Thr, respectively [61]. Activated AKT phosphorylates target proteins on the cell membrane, which then lose their attachment to the cell membrane and enter the cytoplasm [62]. It further implicates the downstream response of the Bcl2 protein family, which regulates anoikis by controlling mitochondrial permeability [63].

### NF-κB signaling pathway

Akt can undergo proteasome-mediated degradation and release NF-κB through IkB kinase (IKK) phosphorylation and inhibition of IκB [64]. IKK supports the translocation of NF-κB to the nucleus by phosphorylating IκB-α, and enhances relA transcriptional activation by phosphorylating the relA activation domain, ultimately upregulating NF-κB target genes Bcl2 and bcl-xl expression, and further triggering anoikis [65].

### Mek/Erk signaling pathway

ECM-cell junctions elevate intracellular ROS levels via integrins, subsequently activating tyrosine kinase Src. Redox regulation of Src mediates integration independent EGFR trans-phosphorylation [66]. Activated Src elicits EGFR downstream signaling (AKT and ERK) in a ligand-independent manner, ultimately leading to Bim downregulation [67]. RAF was identified as the first direct effector of Ras and an upstream kinase of MEK [68]. Ras binds to Raf, and Raf is further phosphorylated to activate ERK1 and ERK2 mitogen-activated protein kinases [69]. Activated ERK1/2 can target mitochondria, enhance ATP synthase activity, maintain mitochondrial membrane potential, inactivate Bad, and reduce cytochrome c release [42]. The second best Ras effector is PI3K, further enhancing the pro-anoikis effect [70].

## Cytoskeleton

Focal adhesion (FA) proteins, which contribute to the establishment and maintenance of integrin-cytoskeleton junctions, can be divided into four categories: (I) integrin-binding proteins that directly bind to actin, such as α-actinin, talin, and fine filament proteins; (II) integrin-binding proteins indirectly associated with the cytoskeleton, including kindlin, core scaffold ILK, plectin, and FAK [71]; (III) non-integrin-binding actin-binding proteins such as nucleoporins; (IV) modulators and signaling molecules that regulate various protein interactions [72]. FA sequesters the BH3 structural domain proteins Bim and Bmf near the membrane. Bmf interacts with dynein light chain 2 (DLC2) of the MYO5/myosin V complex, attaching to actin filaments. Integrin-mediated cell detachment disrupts the actin state, causing Bmf to be released from the cytoskeleton and promoting anoikis [73].

### Protein

#### Bcl-2 protein family

The Bcl-2 protein family includes both pro- and anti-apoptotic members. The BH3-only proteins Bim, tBid and Puma activate Bax and promote anoikis, whereas Bcl-2, Bcl-XL and Mcl-1 suppress Bax activation and anoikis [74]. Mcl-1 is known to prevent anoikis by isolating BH3-only proteins(e.g., Bim and tBid) in mitochondria [75]. Anoikis is highly dependent on the mitochondrial pathway, with Bax serving as a crucial effector of this process [76]. The structure of Bax determines its active state and significantly impacts the intrinsic anoikis pathway [77]. Anti-anoikis agents BCL-2 and BCL-XL sequester BH3 structural domain-only molecules in a stable mitochondrial complex, preventing the activation of Bax and Bak [78]. However, Bad can counteract the anti-anoikis function of Bcl-2 by competing for its BH3 binding domain, indirectly inducing Bax/Bak activation [79]. Bid and Bim directly promote the formation of Bax/Bak oligomers, and the activation of Bax/Bak increases outer mitochondrial membrane permeability, leading to the release of soluble proteins, including cytochrome c, from the intermembrane space into the cytoplasm [80].

### Caspases

Cytochrome c forms apoptosomes by complexing with procaspase-9, apoptotic protease-activating factor-1 (Apaf-1) and dATP [12, 81]. Apaf-1 binding and free caspase-9 maintain a homeostatic equilibrium and activate caspase-9 [82]. Further activation of effector caspases (such as caspase-3, -6 and -7 in mammals) results in the cleavage of specific substrates and promotes cell disassembly [83].

### Anoikis resistance in BC (Fig. 4)

**Fig. 4:**
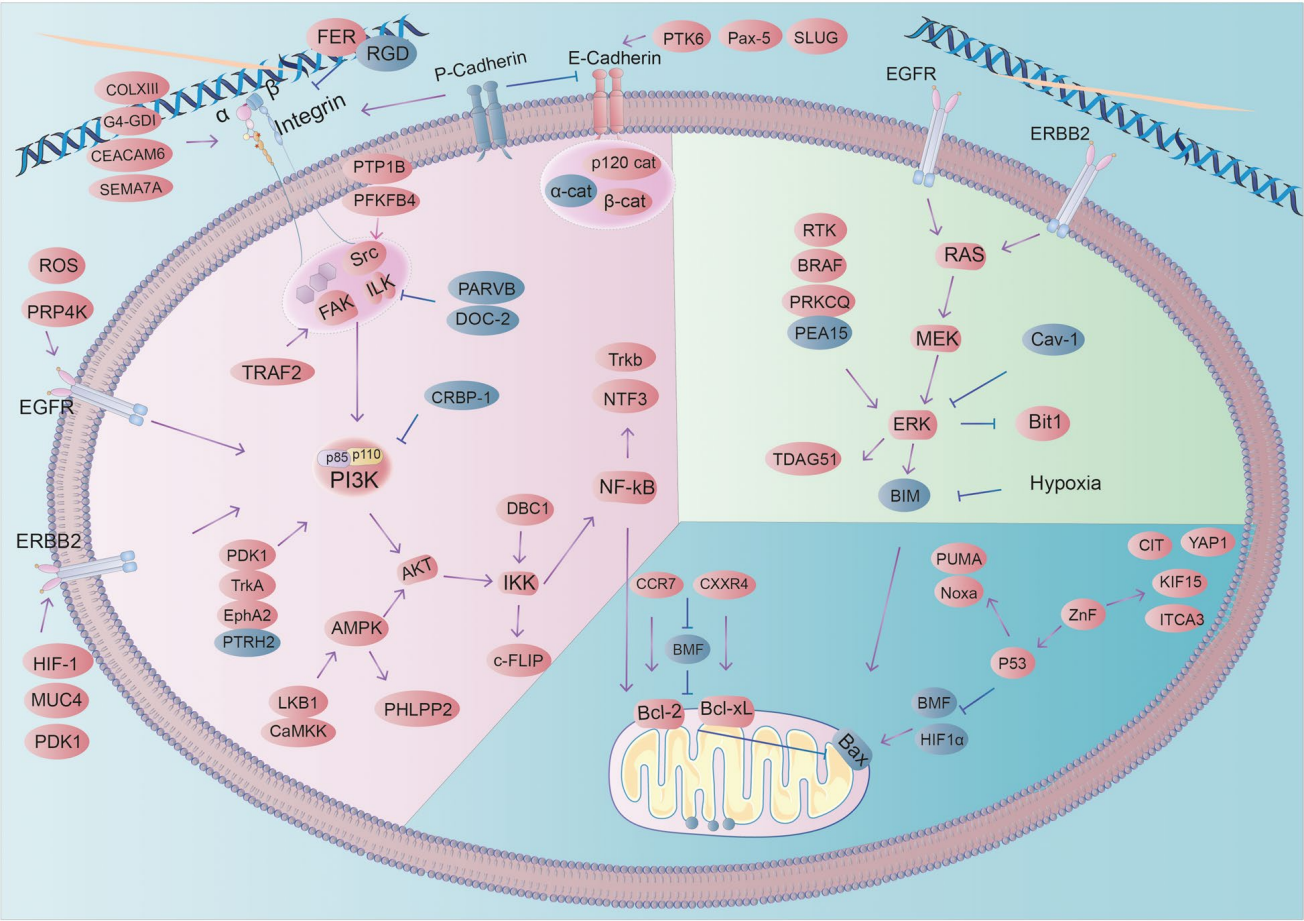
Breast cancer cells detached from tumor tissue have an anoikis-resistant phenotype with multiple mechanisms of altered activation of survival pathways, cytoskeletal remodeling, cellular deformation, and inhibition of mitochondrial apoptotic pathways, resulting in a anoikis-resistant phenotype (red indicates high expression in breast cancer, blue indicates low expression in breast cancer)

**Fig. 5:**
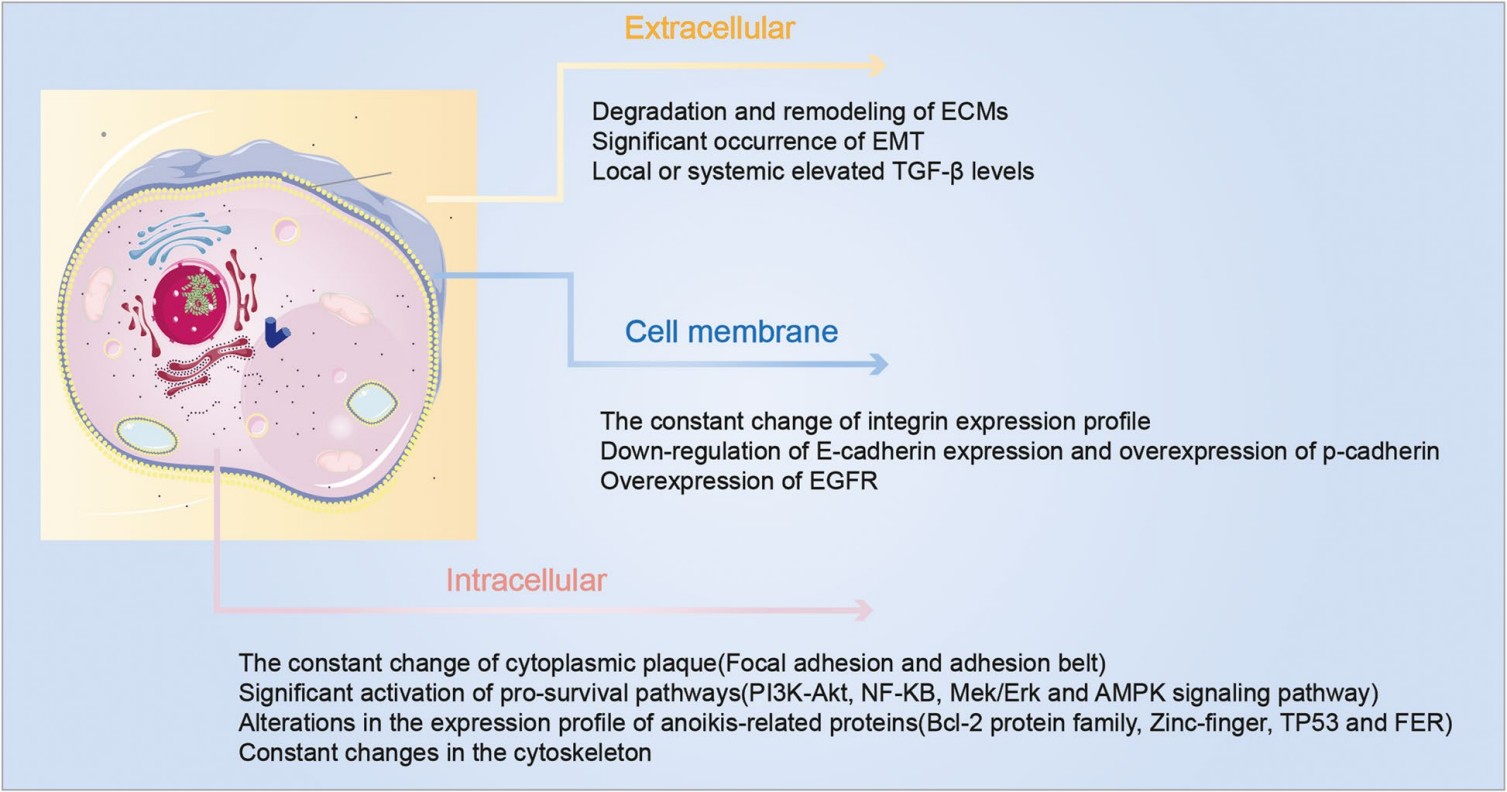
The various factors that contribute to the development of anoikis resistance in BC are outlined

Tumor cells resist anoikis through multiple mechanisms (Fig. 5): extracellular factors such as degradation and remodeling of ECM, significant expression of ECM, and transforming growth factor β; cytosolic factors including continuous alteration of integrins, calmodulin, and altered expression profiles of EGFR and ERBB2; intracellular factors involving the alteration of intracytoplasmic components (e.g., FAK, Src, connexins), abnormal activation of pathways (e.g., PI3K-Akt, Mek/Erk, NF-κB), and expression profiles of anoikis-related proteins (BCL2 family, P53, zinc catalase, ferritin). Additionally, constant changes in the cytoskeleton, miRNA, and reactive oxygen species (ROS) are crucial for tumor cells to counteract anoikis.

### Extracellular

#### ECM: An important tissue barrier for tumor metastasis

ECM is a major component of the tumor microenvironment, regulating numerous pathways in cancer cells, including PI3K/AKT, ERK, Src-FAK, and Rho-GTPases [84]. BC progression necessitates extensive degradation and remodeling of ECM [85]. During tumor progression, stromal EMT deposition increases, altering the chemical composition and mechanical properties of ECM [86]. Cancer cells' invasive capabilities are further enhanced by the mechanical stretching of the collagen matrix or increased matrix stiffness [87].

Invasive BC cells confer anoikis resistance to myofibroblasts during tissue remodeling [88]. Downregulation of tropomyosin-1 (TM1) in BC promotes stress fiber assembly [89], which may disrupt the microfilament structure, thereby enhancing BC cell resistance to anoikis [90].

Abnormal matrix metalloproteinase (MMP) activity is frequently observed in tumors. MMP activation correlates with BC survival signals, with higher MMP activity associated with greater cell migration and metastatic capacity [91]. For example, MMP-11 is overexpressed in many lobular carcinoma cells [92]; MMP-2 is activated on the αvβ3 integrin and its downstream ERK signaling pathway [93]; MMP-7 restores insulin-like growth factor-I (IGF-I) mediated phosphorylation of IGF-IR and activation of Akt [94]; MMP-9 is overexpressed in BC and activates TGF-β/SMAD signaling [95].

### Epithelial-mesenchymal transition (EMT)

EMT enhances metastasis in epithelial carcinomas and leads to cytoskeletal changes that are a prerequisite for the development of anoikis resistance [96]. EMT results in the downregulation of proteins that maintain polarized epithelium, such as occludin, E-cadherin, and claudins, and an increase in mesenchymal proteins (e.g., vimentin, N-cadherin, and smooth muscle actin) [97]. ECM mediates EMT effects through various cell signaling molecules, with Wnt, TGFβ, and Notch ligands playing central roles [98]. Zinc finger E-box binding homeobox 1 (ZEB1) is the master regulator of EMT program [99]. Grainyhead-like2 (GRHL2) and Thyroid Hormone Receptor Interacting Protein 12 (TRIP12) inhibit ZEB1 expression by repressing the ZEB1 promoter and ZEB1 gene, respectively, thereby suppressing TGF-β or Twist-induced or spontaneous EMT [100]. ZEB1 depletion rescues EMT behavior. Loss of CCN6, which is widely found in BC, increases IGF-1 levels in the ECM and activates IGF-1R signaling, leading to EMT [101]. EMT-induced loss of cell polarity in metastatic cancer cells leads to downregulation of the Hippo pathway [102]. The ubiquitin-like modifier-activating enzyme 6 (UBA6) in the human genome initiates ubiquitination through ATP-dependent activation of ubiquitin and inhibits EMT [103]. The tumor protein P53-inducible protein 11 (TP53I11) also inhibites EMT in vitro [104].

### TGF-β is upregulated in BC and promotes a malignant phenotype

TGF-β superfamily members promote advanced cancers while suppressing early events that may lead to cancer [105]. Elevated local or systemic TGF-β levels are typical indicators of metastatic BC and are associated with reduced tumor cell responsiveness to its suppressive function [106]. TGF-β and EGF can synergistically promote malignant phenotypes, such as EMT, anoikis resistance and metastasis, and TGF-β also increases the expression of EGFR [107]. TGF-β is shown to increase the expression of EGFR by stimulating the expression and secretion of multiple ECM components, such as collagen I (COL1A1) and fibronectin (FN1) in stromal fibroblasts, and ECM cross-linking enzymes such as lysyl oxidase (LOX) in BC cells, enhancing the anoikis resistance effect of BC [108].

### Cytomembrane

#### Integrin-ECM interactions promote breast cancer cell survival following matrix stripping

Integrin expression profiles change during cell transformation from normal cells to tumor cells and subsequently to tumor progression [109]. To circumvent anoikis and initiate proliferation, tumor cells must maintain continuous interactions with the extracellular matrix (ECM) through surface receptors such as integrins [110]. Integrin function is influenced by various factors, including the ECM, cell membrane surface receptors, intracellular proteins, and the cytoskeleton.

Collagen XIII, which is highly expressed in breast cancer(BC), induces β1 integrin activation in the ECM [111]. The Rho GTPase regulator D4-GDI, exhibiting increased expression in BC, inhibits β1 expression upon silencing [112]. This leads to Rac1 activation and translocation from cytoplasmic lysates to the cell membrane region, which in turn positively regulates cancer cell migration [113]. α5 is a key signal for Src and ErbB2 conduction in the low-adhesion state and is also an essential mediator for blocking Bim-promoted anoikis [17]. GPI-anchored glycoprotein CEA and carcinoembryonic antigen-related cell adhesion molecule 6 (CEACAM6) are overexpressed in BC [114], mediating their biological effects through enhanced integrin α5β1-fibronectin interactions [115]. The α5β3 integrin specifically recognizes the arginine-glycine-aspartate (RGD) motif in the ECM and is antagonized by the RGD peptide, which holds promise as a drug carrier for targeted therapy in metastatic BC due to its specific recognition of integrins [93]. Loss of Ferritin (FER) on the cell membrane surface increases α6β1 integral protein expression and adhesion of BC cells to collagen I and laminin [116]. Elevated Semaphorin-7a (SEMA7A) protein is observed in BC, promoting anoikis resistance through activating α6β1 integrin and pAKT [117].

### cadherin

#### E-cadherin inhibits tumor metastasis and exhibits downregulated expression in BC

Nearly 90% of infiltrating BC cases demonstrate downregulated or lost E-cadherin expression [118]. Loss of E-cadherin triggers EMT and is accompanied by increased cell motility, invasiveness and resistance to anoikis [119]. Protein tyrosine kinase 6 (PTK6) is expressed in approximately 70% of TNBCs, and its downregulation restores E-cadherin levels [120]. Pax-5, a member of the Paired Box x (Pax) gene family, is commonly expressed in 97% of breast samples. It binds and induces E-cadherin gene expression, inhibiting and reversing EMT in BC [121]. Zinc finger transcription factor (SLUG) is consistently overexpressed in invasive basal BC [122], and also inhibits E-cadherin [123]. E-cadherin cytoplasmic tail protein p120-, α-, γ- and β-linked proteins link cadherins to actin filaments, establishing strong cell–cell adhesion Mutations in these genes often occur in cancer cells, promoting the progression of anoikis resistance [124].

### P-cadherin promotes the progression of BC

P-cadherin is frequently expressed in BC near oxygenated, vascular and hypoxic zones. Its overexpression leads to the activation of FAK, AKT and Src kinases, as well as reduced oxidative stress in stromal isolated BC cells by upregulating carbon flux through the pentose phosphate pathway [125]. P-cadherin activates the heterodimeric α6β4 integrin [125]. Moreover, p-cadherin expression diminishes the invasion inhibitory function of E-cadherin by disrupting the cell membrane E-cadherin/p120-linked protein complexes [126].

### EGFR Overexpression in BC promotes anoikis resistance

EGFR overexpression is observed across all BC subtypes and is higher in the more aggressive TNBC and inflammatory breast cancer (IBC) [127]. EGFR overexpression signals the Mek-Erk pathway and inhibits anoikis by blocking Bim expression [42, 128]. It also indirectly activates Src and EGFR-induced survival signaling due to the dramatically increased ROS levels resulting from the loss of adhesion [129]. Depletion of pre-mRNA processing factor 4 kinase (PRP4K) leads to diminished degradation of EGFR [130]. Recombinant human tyrosine kinase ErbB-2 (also known as HER2 or Neu) stabilizes EGFR in ECM isolated cells by activating the ERK/Sprouty2 (Spry2) pathway [128]. Cells overexpressing EGFR can escape the adjustment of integrin deficiency [131].

ERBB2 is a member of the EGFR family and amplified in 20–30% of human BC [132]. Overexpression of ERBB2 promotes anoikis resistance [42], leading to the filling of the ductal lumen of BC and polar disruption of vesicle-like structures in the breast epithelium [133]. It also simultaneously maintains EGFR expression and EGF-induced signaling allowing cell survival after ECM detachment [17, 42, 134]. Hypoxia-inducible factor-1 (HIF-1) is one mechanism by which cancer cells overexpressing ERBB2 achieve three-dimensional culture growth, anchorage independence and anoikis resistance [135]. ErbB2 driven interferon regulatory factor 6 (Irf6) is downregulated in highly invasive BC cell lines but upregulated in less invasive cell lines, and ErbB2 can downregulate the pro-apoptotic protein Irf6 [136]. Mucin4 (MUC4) interacts directly with the extracellular structural domain of ErbB2 [137], and phosphorylation of ErbB2 is induced by tyrosine residues 1139 and 1248 to isolate ErbB2 and other ErbB receptors, initiating downstream signaling cascades in polarized epithelial cells [138]. PDK1 overexpression significantly enhances the ability of ERBB2 to form tumors [139].

## Intracellular

### Cytoplasmic plaque

#### Focal adhesion-cytoplasmic plaque

Elevated FAK expression in early breast carcinogenesis correlates with poorer OS in BC [140]. Integral protein or FAK activation impedes the ability of death-associated protein kinase (DAPK) to increase p53 levels, which are frequently hypermethylated in DNA and lost in various tumor types [141]. Tumor necrosis factor (TNF) and TNF receptor-associated factor 2 (TRAF2) synergistically function with focal adhesion (FA) signaling, and TRAF2 upregulation in BC [142], promoting cellular resistance to anoikis [143]. Peptidyl-tRNA hydrolase 2 (PTRH2; Bit-1; Bit1) exhibits significant downregulation in advanced BC tissues compared to normal breast epithelial and ductal carcinoma in situ (DCIS) tissues [144]. PTRH2 complexes with FAK at the cytosol to promote PI3K expression, it complexes with AES/TLE to induce anoikis and inhibit EMT [145].

Proto-oncogene c-Src is widely overexpressed in BC [146], promoting anoikis resistance in HER2 + BC cells in an integrin-dependent manner [17]. Protein tyrosine phosphatase 1B (PTP1B) levels increase significantly in BC tissues [147], and PTP1B is the primary phosphatase that dephosphorylates c-Src, thereby controlling its kinase activity in BC cell lines [148]. The metabolic enzyme 6-phosphofructo-2-kinase/fructose-2,6-bisphosphatase 4 (PFKFB4) is overexpressed in BC [149], and activates src-3, which in turn activates the estrogen receptor (ER) [150].

ILK expression is upregulated in human tumors and tumor cell lines [151], and its function is required for TGFβ-1-induced EMT in mammary epithelial cells [152]. β-parvin specifically binds ILK [153], is down-regulated in BC [154], and its overexpression inhibits ILK kinase activity [154]. Rictor, a component of the mTORC2 complex, regulates ILK's ability to promote Akt phosphorylation [155]. DOC-2/hDab2 (DOC-2) inhibits ILK activity through an Akt-independent pathway [156].

### Adhesion belt-cytoplasmic plaque

In over 50% of BC, β-catenin signaling is upregulated through multiple pathways [157]. Stable β-catenin induces mammary tumorigenesis in mice, functioning as a crucial part of classical Wnt signaling and binding to the cytoplasmic tail of E-cadherin, thereby negatively regulating E-cadherin [158]. β-catenin directly interacts with c-erbB-2 protein and EGFR, playing a crucial role in the tumor signaling pathway [159]. Cadherin system inactivation may increase β-catenin, which activates the Wnt signaling pathway [160]. β-Catenin target genes cyclin D1 and c-myc are also upregulated [161].

P120-catenin is reduced or lost in early BC [162], but it accumulates in the cytoplasm and nucleus rather than being degraded [163]. The transition from a complete loss of protein expression to accumulation is a significant characteristic of p120-catenin. Loss of E-cadherin leads to reduced adhesion and p120-catenin translocation to the cytoplasm and nucleus. P120-catenin may function as a tumor suppressor or metastasis promoter [164], displaying full oncogenic properties in the absence of anchoring [165]. p120-catenin can indirectly activate the Rho/Rock signaling pathway by binding to the Rho function inhibitor Mrip [58]. It can also directly bind RhoA and act as a Rho-GDP dissociation inhibitor (RhoGDI), thereby inhibiting stress fiber-mediated contractility and increasing tumor cell motility [166]. Nuclear p120-catenin controls the anchorage-independent upregulation of Wnt11 by repressing Kaiso-mediated transcription, thereby promoting RhoA activation [167].α-catenin inhibits tumor progression [168], and is downregulated in BC with a more metastatic phenotype [169]. Its loss can lead to lower BC survival rates [170]. α-catenin controls formic acid-dependent radial actin filament formation [171] and also competes with the Arp2/3 complex to bind actin, thereby inhibiting actin branching [172]. Additionally, α-catenin enhances the binding of p120-catenin to E-cadherin, making the connection more stable. The loss of α-catenin leads to mislocalization and aggregation of E-cadherin, which predominantly remains in the plasma membrane [173]. Deletion of α-catenin activates both Rho- and Rock-dependent actin contraction [58]. Furthermore, α-catenin inhibits tumorigenesis by interacting with IκBα [169].

### Signaling pathway

#### Alterations in the PI3K-Akt signaling pathway are prevalent in BC

Gene amplification encoding PI3K or Akt, or mutations in pathway components, may result in constitutive activation of the PI3K-Akt pathway. Approximately 25% to 30% of BC cases contain PIK3CA mutations [174].

Cellular Retinol Binding Protein 1 (CRBP-1) suppresses the PI3K/Akt survival pathway in a retinoic acid receptor-dependent manner. CRBP-1 downregulation is associated with a malignant phenotype in BC, and CRBP-1 inhibits p85 phosphorylation at Y688 [175]. A series of proteins with increased expression in BC, including γ-catenin, PDK1, tyrosine protein kinase receptor A (TrkA), and Ephrin type-A receptor 2 (EphA2), positively regulate the PI3K-AKT pathway and impair anoikis function in BC [176]. Overexpression of PDK1 protein and its mRNA, recruited by PIP3, elevates PDK1 gene copy number, and enhances AKT and downstream pathway activity [139, 177]. TrkA protein is overexpressed in a large cohort of clinically relevant BC [177]. TrkA and downstream AKT signaling modulate cell growth and viability [178]. EphA2, a receptor tyrosine kinase and guanine nucleotide exchange factor for GTPase RhoG, interacts with Ephexin4 to activate RhoG and PI3K downstream of EphA2 [179].

Phosphatase and tensin homologs (PTEN) absent on chromosome 10 are frequently mutated in BC, inhibiting Akt activation [180]. PTEN interacts with FAK, reducing FAK phosphorylation and activity [181]. Heterozygous deletion of PTEN activates the PI3K/Akt and MAPK pathways, while pure deletion of PTEN expression results in the activation of both, thus conferring BC anoikis resistance [182]. WNT5A, a member of the WNT family, exhibits down-regulated in BC expression and inhibits ERK1/2 activity in BC cells [183].

### NF-κB Signaling expression is significantly upregulated in BC

I-κB kinase (IKK), a core regulator of NF-κB signaling, is associated with tumorigenesis through its three isoforms (α, β, and ε) [184]. The Deleted in Breast Cancer 1 (DBC1) protein interacts with IKK-β as a cofactor, stimulating the phosphorylation of nuclear relA (serine-536), promoting transcriptional activation of NF-κB target genes, and upregulating the expression of target genes that protect against anoikis (e.g., c-FLIP and Bcl-xl) [65, 185]. IKKε is amplified and overexpressed in approximately 30% of BC cases [186], and activates classical NF-kB signaling by directly phosphorylating the RelA/p65 subunit [187].

In BC cells detached from the ECM, NF-κB activity was found to be increased sixfold compared to normal breast cells. RelA and NF-κB1 are transcription factors responsible for upregulating neurotrophic receptor TrkB and its ligand neurotrophic factor 3 (NTF3) [188]. Etoposide induced 2.4 (EI24) attenuates NF-κB activity by binding to complex I component TRAF2 and inducing its lysosome-dependent degradation. EI24 has been identified as an oncogene and significantly downregulated in invasive cancers [189].

### MEK/ERK Signaling pathway is significantly activated in BC

ERK signaling activation is observed in more than 85% of cancers and is directly attributable to genetic alterations in its upstream activators, including BRAF, Ras, and RTK [190]. Ras-related protein Rab25 activates intracellular signaling pathways, and Rab25 knockdown reduces phospho-ERK1/2 levels and promotes BC cell proliferation [191]. Multiple Ras signaling pathways contribute to breast EMT, with the ERK signaling pathway potentially being a key component downstream of EGFR activation during tumorigenesis [192].

Protein kinase C theta (PRKCQ/PKCθ) activates ERK in a kinase activity-dependent manner [193]. Sustained ERK signaling activation upregulates T-cell death-associated gene 51 (TDAG51), which inhibits ERK-mediated mammary EMT [194]. Reduced phosphoprotein expression in astrocytes-15 (PEA15) in metastatic BC cells leads to ERK1/2 binding and altered ERK1/2 cellular localization and target preference [195]. Caveolin-1 (Cav-1) expression is downregulated in BC, inhibiting stromal invasion and blocking laminin-dependent activation of ERK1/2 [196].

Upon ECM attachment loss, p38ERK is activated and recruited into the high molecular weight mitochondrial complex, a necessary signal for Bax translocation to the mitochondria and Bax activation. P38ERK targeting the outer mitochondrial membrane (OMM) drives anoikis [197]. ECM attachment loss also results in the release of PTRH2 from mitochondria into the cytoplasm. Increased ERK levels and decreased ERK-directed phosphatase activity disrupt mitochondrial Bit1 [144], and loss of Bit1 expression leads to increased ERK activation [145, 198]. Bit1 expression was reduced in higher grade BC with positive lymph node metastases, compared to normal breast tissue or invasive low BC [145].

### AMPK Signaling pathway is actively expressed in BC

Stromal deprivation activates AMPK activity through LKB1 and Ca2 + /cadherin-dependent protein kinase kinase (CaMKK) [199], and further by upregulating the Akt phosphatase pleckstrin-homology (PH)-domain leucine-rich-repeat protein phosphatases (PHLPP2) while inhibiting AKT activity. PAMPK high/pAkt low state impairs both autophagy and metastasis [200]. AMPK directly affects metabolic enzymes (such as G6PD, ACC, and HMG-CoA reductase) and plays a key role in regulating growth and metabolism [201]. AMPK contributes to anti-anoikis by phosphorylating phosphoprotein 15 kDa (PEA15) and inhibiting mTORC1 [202] as well as Acetyl-CoA carboxylase (ACC) to maintain NADPH homeostasis [199].

## Protein

### Bcl-2 Protein Family Alterations Inhibit Anoikis

Expression of several pro-apoptotic BH3-only proteins of the Bcl-2 family is downregulated following ECM detachment, resulting in an intrinsic cell death cascade. Hypoxia contributes to blocking the expression of pro-apoptotic BH3 family members, such as Bim and Bmf, in epithelial cells [203]. Chemokine receptors CCR7 and CXCR4 are highly expressed in BC [204]. Activation of CCR4 and CCR7 by their respective chemokine ligands CXCL12, CCL19/CCL21 downregulates pro-apoptotic Bmf and upregulates pro-survival proteins Bcl-2 and Bcl-xL, specifically reducing the sensitivity of metastatic BC cells to anoikis [205].

### Zinc-finger protein binds specifically to DNA, RNA, and DNA-RNA sequences to inhibit anoikis

ZNF367 is significantly upregulated in BC, interacts with the chromatin remodeling protein Brahma-related gene 1 (BRG1), and transcriptionally activates Citron (CIT) and TP53. It represses the Hippo pathway and activates Yes-associated protein 1 (YAP1), causing anoikis resistance [206]. ZnF protein E4F transcription factor 1 (E4F1) is involved in DNA repair, with E4F1 binding to the catalytic subunit BRG1/SMARCA4 and mediating its recruitment to DNA damage together with Poly(ADP-ribose) (PAR) polymerase-1 (PARP-1) [207]. ZNF367 is an upstream transcription factor of kinesin superfamily proteins 15 (KIF15), highly expressed in BC tissues and regulates mitosis during cellular processes [208]. Additionally, the expression of integrin α 3 (ITGA3) is also regulated by ZNF367 [209].

### Tumor protein p53 (TP53) is significantly altered in BC

TP53 can mediate anoikis by upregulating the transcription of pro-apoptotic Bcl-2 family members, including PUMA, Noxa and Bax [210]. TP53 is highly mutated in BC [211], with mutant p53-R273H inhibiting BMF via AKT [212], and mutant p53P151S causes increased tumor growth and metastasis [213]. TP53-inducible protein 11 (TP53I11) is a transcriptional target of TP53. Deletion of TP53I11 promotes AKT/m-TOR and AMPK pathways, respectively, subsequently reducing m-TOR activation and consequently maintaining energy homeostasis in BC cells [214], promoting cell survival after ECM isolation. HIF1α is a key mediator of cellular adaptation to hypoxia [215], and TP53I11 overexpression inhibits HIF1α expression and affects EMT and metastasis [104]. YAP positively regulates p53 family members, YAP protein is significantly reduced in BC, and knockdown of YAP short hairpin RNA inhibits anoikis [216]. Transcription factor p73 is a homolog of p53 and can be expressed as a pro- or anti-apoptotic isoform. Conversely, p73 is stably expressed in cancer and can replace defective p53, inducing anoikis [217].

### Ferritin (FER) promotes BC cell migration

FER protein is significantly elevated in metastatic BC cells and is closely associated with the development of anoikis resistance mechanisms [116]. FER promotes BC cell migration and inhibits anchorage-dependence, leading to increased formation of distant metastases [218]. The primary mechanism of action involves inhibiting cellular morphological changes, including the regulation of actin stress fiber and focal adhesion formation [219]. FER deficiency increases α6β1 integrin expression and collagen I(COL1) adhesion and laminin in BC cells [220]. Consequently, FER controls the recirculation of α6-integrins in metastatic BC cells [116], and downregulates the synthesis of laminin-binding glycans, reducing cell adhesion to laminin [221].

### Abnormal actin presence as a main feature of malignant tumors

Cytoskeletal proteins induce anoikis in BC cells. Restoration of stress fiber networks can be achieved by enhancing the expression of key cytoskeletal proteins, thereby regulating focal adhesion activity and sensitizing tumor cells to anoikis [89]. Osteopontin (OPN) is highly expressed in various cancers and increases the potential for cell invasion. Bone bridging proteins have two forms corresponding to two functions: the soluble form favors invasiveness, and the aggregated form favors adhesion [222]. KIAA0100 protein is upregulated in BC and acts as a microtubule binding protein to stabilize the MT structure [223]. KIF18A is overexpressed in human BC. The inhibition of Kif18A stabilizes MT at the leading edge, inducing inactivation of the PI3K-Akt signaling pathway, reducing cancer cell migration and proliferation [224]. High expression of ubiquitin-binding enzyme E2S (UBE2S) in BC disrupts the actin cytoskeleton [225]. BC metastasis suppressor 1 (BRMS1) decreases cytoskeletal reorganization and cell adhesion protrusion formation [226]. Histone deacetylase 6 (HDAC6) is located in the cytoplasm, upregulated in multiple cancers, binds to MT and the actin cytoskeleton, and regulates cell adhesion [227].

### Gene localization analysis

**Fig. 6:**
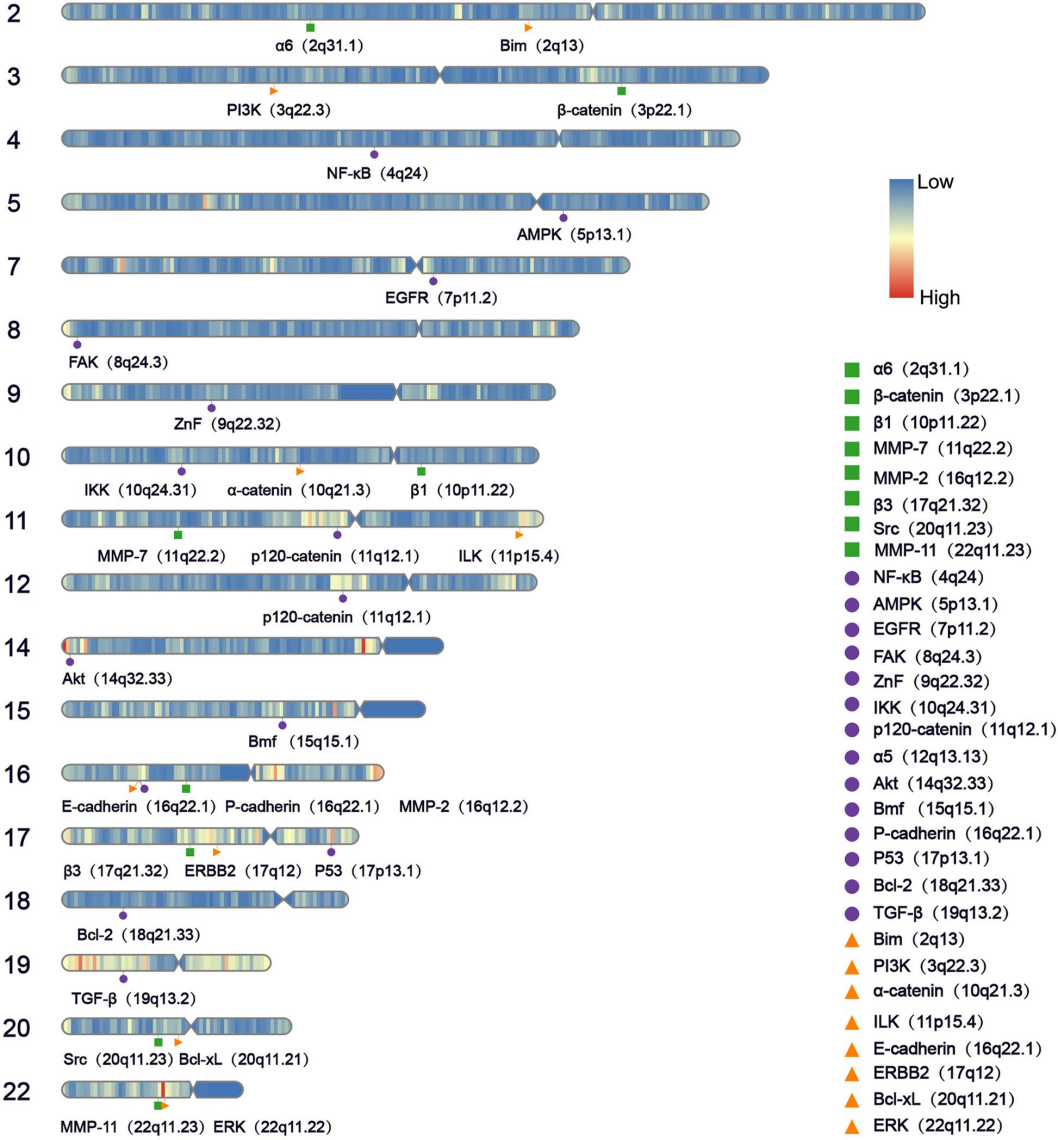
Chromosomal location of the major associated proteins of anoikis resistance in BC. Blue indicates low density of gene distribution, red indicates high density of gene distribution

The major structures and components of BC anoikis resistance were targeted, with a focus on studying their loci on chromosomes (Fig. 6). We aimed to find out if there are genes at similar loci undergo cascading alterations and therefore affect BC anoikis resistance. This analysis was conducted by accessing the NCBI (National Center for Biotechnology Information; https://www.ncbi.nlm.nih.gov/) database, obtaining gene locus information, including chromosome numbers and the starting and ending points of the genes on the chromosomes, and using the "RIdeogram" R package to map the loci for presentation. According to our results, E-cadherin and P-cadherin were located at 16q22.1, and P-cadherin was located in the upstream of E-cadherin [228]. Long-arm heterozygous deletion (LOH) of chromosome 16 occurs in at least half of breast tumors, and the smallest region of overlap (SRO) is found in the region of 16q22.1 [229], suggesting the presence of tumor suppressor genes (TSG) in this region of the BC. E-cadherin fits this profile [230], while P-cadherin is associated with poor survival [231]. Further exploration is warranted to determine if cascade alterations occur at the genetic level. Similarly, MMP-11 (22q11.23) and ERK (22q11.22), both located on chromosome 22q11, are highly expressed in BC. Copy number gains on BC chromosome 22q11 were more frequent in the Chinese cohort than in the Cancer Genome Atlas [232], and gains in the 22q11.2 region were shown to contribute to triggering EMT. The summary of genetic loci associated with BC anoikis resistance provided here is intended to inform studies of metastatic BC at the chromosomal level.

### Micro RNA (miRNA)

MicroRNAs (miRNAs) are small non-protein-coding RNAs, approximately 18 to 25 nucleotides in length, that are predicted to regulate the expression of over 90% of genes at the post-transcriptional level. These miRNAs influence various cellular and molecular processes, including anoikis, angiogenesis, tumorigenesis, tumor growth, cell migration, metabolic pathways, and signal transduction [233]. The results (Table 1) indicate that miR-630, miR-223, miR-200c, miR-148b, miR-18a, miR-124, miRNA-200b, miR-491-5p, miR-6744-5p, and miRNA-188-3p were down-regulated in BC. Conversely, miR-23b, miR-27b, miR-522, miR-200a, and miR-181a were up-regulated in BC, leading to enhanced anoikis resistance.

**Table 1:** MicroRNAs associated with anoikis in BC

Role	miRNA	MCF-7	MDA-MB-231	Target Points	Function	Refer-ences
Promotes	miR-630	Down	Down	Insulin like growth factor 1 receptor (IGF1R)	MiR-630 directly regulates IGF1R, which subsequently leads to a decrease in HER2 and EGFR, promotes anoikis	[234]
Promotes	miR-223	Down	Down	Caprin-1	MiR-223 targets the 3'UTR of Caprin-1 miRNA and downregulates Caprin-1 expression, promotes anoikis	[235]
Promotes	miR-200c	Down	Down	Zinc finger E-box binding homeobox (ZEB1和ZEB2)	MiR-200c downregulates the expression of E-cadherin and other epithelial polar and functional mediators to maintain the mesenchymal phenotype, promotes anoikis	[236]
Promotes				FN1、Moesin (MSN), leptin receptor (LEPR) and Rho GTPase activating protein 19 (ARHGAP19)	MiR-200c maintains the epithelial phenotype by repressing mesenchymal and neuronal gene programs involved in cell motility and anoikis resistance	[237]
Promotes				TrkB and NTF3	MiR-200c targets the NF-κB upregulated TrkB/NTF3 autocrine signaling loop to enhance anoikis sensitivity in triple-negative BC	[188]
Promotes	miR-148b	Down	Down	cathepsin A (CTSA), neuropilin 1 (NRP1), colony stimulating factor 1 (CSF1), MMP15 and integrin subunit alpha 5 (ITGA5)	MiR-148b directly targets participants in integrin signaling, such as ITGA5, ROCK1, PIK3CA/p110α, and NRAS, as well as CSF1, promotes anoikis	[238]
Promotes	miR-18a	—	—	hypoxia-inducible factor 1 (HIF1A)	MiR-18a promotes anoikis via the HIF1A-dependent pathway	[239]
Promotes	miR-124	Down	Down	connective tissue growth factor (CTGF), ras homolog gene family member G (RhoG), integrin-β1 (ITGB1), Rho-associated coiled-coil containing protein kinase 1 (ROCK1) and Cyclin-dependent kinase 6 (CDK6)	MiR-124 acts through a mechanism that down regulates CTGF, ITGB1, RhoG and ROCK1. CTGF, RhoG, ITGB1 and ROCK1 are upregulated in BC, and Overexpression of these proteins was positively correlated with anoiks resistance and poor prognosis	[240]
Promotes	miR-200b	Downregulated in BC cells that acquire a metastatic phenotype		Pin1	MiRNA-200b regulates the activity of PEA3 and ELK-1 through the Pin1-pERK pathway, forming a self-regulatory feedback loop, promotes anoikis	[241]
Promotes	miR-491-5p	Down	Down	ZNF-703	MiR-491-5p suppresses BC metastasis by targeting ZNF-703 to regulate AKT/mTOR pathway, promotes anoikis	[242]
Promotes	miR-6744-5p	Down	Down	N-acetyltransferases 1 (NAT1)	MiR-6744-5p directly targets and downregulates NAT1, which induces DNA damage through chemical carcinogenesis and inhibits anoikis by suppressing reactive oxygen species (ROS)	[243]
Promotes	miRNA-188-3p	Down	Down	Transmembrane p24 trafficking protein (3TMED3)	MiRNA-188-3p is a novel negative regulator of TMED3 that slows the proliferation, migration and invasion of BC cells, promotes anoikis	[244]
Suppresses	miR-23b	Up	Up	Nischarin (NISCH)	Knockdown of miR-23b and miR-27b enhances NISCH expression, and NISCH inhibits NF-κB phosphorylation, suppresses anoikis	[245]
Suppresses	miR-27b					
Suppresses	miR-522	Up	Up	ETS transcription factor1 (ELK1)和 E2F transcription factor 3 (E2F3)	Overexpression of miR-522 significantly downregulates ELK1 and E2F3, leading to loss of adhesion and G1/S phase block, suppresses anoikis	[246]
Suppresses	miR-200a	Up	Down	Yes-associated protein 1 (YAP1)	MiR-200a directly binds to the 3'-UTR of YAP1 to reduce YAP1 expression, and downregulates YAP1 to inhibit the expression of caspase-3 division and pro-anoikis proteins (Bax and Bim)	[247]
Suppresses	miR-181a	Expression of miR-181a is upregulated by TGF-β		Bim	The high expression of miR-181a suppressed the expression of Bim. miR-181a/b was also shown to regulate Bcl-2, ATM, p27, K-Ras and TIMP3, suppresses anoikis	[248]

### Targeted therapy

Targeted therapy for BC is essential in the treatment of this condition.Controlling BC in situ and eliminating the possibility of metastasis serve as effective strategies for improving treatment outcomes. Restoring BC cell anoikis sensitivity can help achieve this objective. A collection of drugs and compounds reported to influence BC anoikis function has been compiled, including plant extracts and drugs commonly used in clinical practice for other diseases, in addition to conventional BC targeted therapies. These are all summarized in Table 2. Among the compiled results, Goniothalamin (GTN) appears to be the most comprehensive drug for targeting the restoration of anoikis function. Further exploration is required to determine its potential for treating BC.

**Table 2:** Targeted agents associated with anoikis in breast cancer

Drugs/Compounds	Type	Functions	References
Ouabain	T47D	Activating the mitochondrial cystathionase pathway triggers anoikis	[249]
TAK-242	MDA-MB-231, MCF-7, SKBR3, BT-474	Increasing anoikis by altering NF-кB and p53 related apoptosis genes in BC cells	[250]
SAHA and TRAIL	MDA-MB-231, MCF-7	Activating caspase-3, phosphorylation of EGFR, reduction of phosphorylated ERK1/2, induction of reduction of Phosphorylated BimEL, and increase of BimEL dephosphorylated form, promoting anoikis	[251]
Berberine	MDA-MB-231, MCF-7	Inducing cell cycle arrest to promote anoikis resistance	[252]
4-methylumbelliferone (4-MU)	MDA-MB-231, MCF-7	Regulating hyaluronic acid/HAS2/CD44 and specific matrix effectors, increasing anoikis	[253]
Curcumol	MDA-MB-231, IV2	Regulating the YAP1/Skp2 molecular pathway, promoting anoikis	[254]
isoliquiritigenin	MDA-MB-231, BT-549	Down–regulating COX-2 and CYP4A signaling; decreasing the expression levels of phospho-PI3K (Tyr(458)), phospho-PDK (Ser(241)) and phospho-Akt (Thr(308)), promoting anoikis	[255]
archazolid	MDA-MB-231, 4T1, T24, 5637	Reducing expression of c-FLIP, activation of caspase-8, and early increase in active integrin-β1 and pro-anoikis protein BIM	[256]
pygenic acid A (PA)	MDA-MB-231, 4T1	Down-regulating pro-survival proteins such as cIAP1, cIAP2 and survival; down-regulating anoikis resistance proteins such as p21, cyclin D1, p-STAT3 and HO-1	[257]
Disulfiram (DSF)	MDA-MB-231, 4T1	Inhibiting focal adhesion, regulating waveform proteolysis, and activating calpain, promoting anoikis	[258]
pretubulysin	MDA-MB-231, 4T1	Activation of mitogen-activated protein kinases (especially JNK (c-Jun N-terminal kinase)) and phosphorylation of Mcl-1, induces proteasomal degradation of Mcl-1, promoting anoikis	[259]
Epoxyazadiradione	MDA-MB-231	Reducing EGFR expression, promoting anoikis, inhibits colony formation, downregulating MMP-9 and fibronectin, induces G2/M phase block, downregulating cyclin A2/cdk2, interfering with cellular metabolism and inhibiting nuclear translocation of nuclear factor NF-kB	[260]
Tubeimoside V	MDA-MB-231	Inhibition of non-anchored culture-induced CAV-1 overexpression, EGFR activation, and ITGB1-FAK activation, promoting anoikis	[261]
Goniothalamin(GTN)	MDA-MB-231	GTN reverses EMT, inhibits EGFR, FAK and Src pathways, reduces matrix metalloproteinase secretion, increases E-cadherin protein and decreases N-cadherin levels, promoting anoikis	[262]
HPW-RX40	MDA-MB-231	Inhibiting integrin expression and activation and shedding-induced FAK activation and downstream phosphorylation of Src and pilein, promoting anoikis	[263]
Ziyuglycoside II	MDA-MB-231	Regulating Src/EGFR-dependent ITGB4/FAK signaling pathway, promoting anoikis	[264]
Salinomycin	MDA-MB-231	Inhibiting STAT3 activation and reduces CD44 + /CD24- stem cell-like populations, promoting anoikis	[265]
Dieldrin	MDA-MB-231	Increasing resistance to anoikis and TrkB expression	[266]
Metformin and 2-deoxy glucose	MDA-MB-231	Strong activation of AMPK increases phosphorylation of ACC, which increases the G2/M phase cell population and triggers G2 and mitotic (M) phase arrest of the cell cycle, promoting anoikis	[267]
5-azacytidine (5-AzaC)	MCF-7	Nucleoside analogs that reduce DNA methylation, induce anoikis, inhibit mammosphere formation and reduce metalloproteinase 9 activity, promoting anoikis	[268]
Pt(O,O'-acac)(gamma-acac)(DMS)	MCF-7	Activating PKC-α, which generates ROS, leads to increased Ca(2 +) permeability and decreased PMCA activity, promoting anoikis	[269]
BKM120 Joint ATO	MCF-7	BKM120 enhances ATO-induced anti-proliferative effects by inducing G1 phase block and reducing DNA synthesis in BrdU-treated cells, promoting anoikis	[270]
Benzothiazole carbamates and amides	MCF-7	Inducing anoikis, G2/M cell cycle arrest and reducing ROS levels	[271]
PS, JCP1 and JCP2 extracts	MCF-7	Inhibition of β1-integrin expression, F-actin, cell detachment, promoting anoikis	[272]
Pt(O,O'-acac)(gamma-acac)(DMS)	MCF-7	Inducing anoikis and alters cell migration, anchorage independence, matrix interactions, and MMP activity	[273]
2-Deoxy-D-Glucose	Hs578T, Hs578Ts(i) 8	Decreasing oxidative phosphorylation, increase glycolysis, and inhibit tumor migration and invasion, promoting anoikis	[274]
Aspirin	4T1, E0771	Aspirin targets thromboxane A2 (TXA2), TXA2 receptor (TP) and thromboxane A2 synthase 1 (TBXAS1) are upregulated in metastatic BC cells and confer anoikis resistance through sustained activation of Akt	[275]
Monascin (MS)	4T1	Inhibiting E-cadherin and β-catenin expression in cells, promoting anoikis	[276]

## Conclusion and outlook

Metastasis is a complex biological process and the leading cause of death in BC patients. Tumor cells detach from their original locations, migrate, invade the circulatory and lymphatic systems, and ultimately invade and colonize distal locations, growing and proliferating to form secondary tumors. The ability of cells to survive independently is a critical factor in the metastatic process, as tumor cells with malignant potential can initiate mechanisms to resist anoikis and survive. Cancer cells undergo alterations at various levels, including genes, proteins, cytoskeleton, and cell structure, in order to resist anoikis. Different cancer cells are involved in distinct mechanistic alterations, and multiple mechanisms often collaborate to affect a single cell. The cumulative result of these factors presents a significant challenge for clinical treatment.

Precisely targeting the critical period when anoikis resistance occurs (i.e., after disruption of tumor epithelial cell-ECM interactions and before metastatic spread) is an essential approach for prognostic regression of BC. Current strategies using anoikis sensitizing drugs primarily involve combinatorial algorithms aimed at reducing the activation of alternative signaling pathways and maximizing efficacy. The mechanism by which GTN promotes anoikis in multiple directions requires further investigation.

Promoting the restoration of the anoikis phenotype in tumor cells through modulation of miRNAs is another highly promising approach. MiRNAs can play a role in the clinical treatment of patients with tumor metastasis, in addition to their previous value as predictive molecular markers in clinical work. Consequently, potential anoikis sensitizing drugs and genetic manipulation targeting miRNA families could provide a practical framework for inhibiting tumor metastasis.

## Data Availability

Not applicable.

## Abbreviations

BC: Breast Cancer
HER2: Human Epidermal Growth Factor Receptor2
ECM: Extracellular Matrix
PPP: Pentose Phosphate Pathway
BMP: Bone Morphogenetic Protein
GFRs: Growth Factor Receptors
FAK: Focal Adhesion Kinase
JAK: Janus-activated Kinase
PI3K: Phosphatidylinositol 3-kinase
IMS: Intermembrane Space
KD: Kinase Domain
PDK1: 3-Phosphatidylinositol-dependent Protein Kinase 1
FA: Focal Adhesion
TNFR2: Tumor Necrosis Factor Receptor 2
Apaf-1: Apoptotic protease-activating factor-1
MMP: Matrix Metalloproteinase
ZEB1: Zinc finger E-box Binding homeobox 1
GRHL2: Grainyhead-like2
TP53I11: Tumor Protein P53-inducible protein 11
FN1: Fibronectin
CEACAM6: Carcinoembryonic Antigen-related Cell Adhesion Molecule 6
FER: Ferritin
IARC: International Agency for Research on Cancer
TNBC: Triple Negative cancer
COLI: Collagen I
ROS: Reactive Oxygen Species
CAM: Cell Adhesion Molecules
ILK: Integrin-related Kinase
TEB: Terminal Bud
PLCγ: Phospholipase Cγ
EGFR: Epidermal Growth Factor Receptor
PH: Pleckstrin Homology
PIP3: Phosphatidylinositol 3,4,5-trisphosphate
IKK: IkB kinase
TNF: Tumor Necrosis Factor
DLC2: Dynein Light Chain 2
TM1: Pro-myosin-1
IGF-I: Insulin-like Growth factor-I
UBA6: Ubiquitin-like Modifier-activating Enzyme 6
TRIP12: Thyroid Hormone Receptor Interacting Protein 12
LOX: Lysyl Oxidase
RGD: Arginine-glycine-aspartate
SEMA7A: Semaphorin-7a
